# HEPATIC AGO2-MEDIATED RNA SILENCING REGULATES SYSTEMIC GLUCOSE METABOLISM.

**DOI:** 10.1093/geroni/igz038.3240

**Published:** 2019-11-08

**Authors:** Kazutoshi Murakami

**Affiliations:** ​Kurashiki Central Hospital, Kurashiki, Okayama, Japan

## Abstract

Obesity causes various metabolic complications, including insulin resistance and T2DM. However, currently, we have a limited understanding of the pathophysiology in the development of these processes. It’s generally considered that obesity develops when energy intake chronically exceeds total energy expenditure. The liver is a major organ for energy consumption, then mRNA translation accounts for the majority of energy expenditure in liver. While RNA silencing regulates mRNA translation, it’s unclear if RNA silencing regulates glucose metabolism. To investigate the role of RNA silencing in glucose metabolism, we focused on Argonaute 2 (Ago2), which is the main component of RNA-induced silencing complex that carries out RNA silencing. By generating liver-specific Ago2-deficient (L-Ago2 KO) mice, we revealed Ago2 regulates the maturation process of metabolic disease related miRNAs (MD-miRNAs), that silence genes critical for glucose metabolism. In addition, Ago2-deletion enhances ATP consumption associated with mRNA translation. Consequently, inactivation of hepatic Ago2 protect from diet-induced glucose intolerance in mice. Then, to further investigate if these molecular mechanisms are still activated in “older mice”, we employed 34-week of age mice and analyzed. Around this age, despite having the similar body weight, L-Ago2 KO mice fed HFD exhibited lower blood glucose and serum insulin levels. Consistently, expression levels of MD-miRNAs were decreased in the liver of L-Ago2 KO mice fed HFD. These results suggest that hepatic Ago2 function is continuously activated in “older mice” fed HFD, leading to enhanced biogenesis of MD-miRNAs and reduction of their target mRNAs, and these alterations are associated with systemic glucose metabolism.

